# Increased Expression of Neurotrophin 4 Following Focal Cerebral Ischemia in Adult Rat Brain with Treadmill Exercise

**DOI:** 10.1371/journal.pone.0052461

**Published:** 2013-03-18

**Authors:** Jin-Young Chung, Min-Wook Kim, Moon-Suk Bang, Manho Kim

**Affiliations:** 1 Department of Neurology, Seoul National University Hospital, Chongno-ku, Seoul, Korea; 2 Department of Rehabilitation Medicine, College of Medicine, The Catholic University of Korea, Seoul, and Institute of Catholic Integrative Medicine (ICIM), Incheon St. Mary's Hospital, Incheon, South Korea; 3 Department of Rehabilitation Medicine, Seoul National University Hospital, Chongno-ku, Seoul, Korea; University G. D'Annunzio, Italy

## Abstract

Neurotrophin 4 (NT-4) belongs to the family of neurotrophic factors, and it interacts with the tyrosine kinase B (trkB) receptor. NT-4 has neuroprotective effects following cerebral ischemia. Its role might be similar to brain-derived neurotrophic factor (BDNF), because both interact with trkB. Exercise also improves neural function by increasing neurotrophic factors. However, expression profiles of NT-4 in the brain during exercise are unknown. Here, we assessed the expressions of NT-4 and its receptor, trkB, following cerebral ischemia and hypothesized that exercise changes the expressions of NT-4 and trkB. Results showed that in a permanent middle cerebral artery occlusion rat model, ischemia decreased NT-4 and trkB expression. Immunohistochemistry showed their immunoreactivities around the region of the ischemic area. Treadmill exercise changed the expression of NT-4, which increased in the contralateral hemisphere in rats with ischemic injury. TrkB also showed similar patterns to its neurotophins. The change in NT-4 suggested that exercise might have primed NT4 production so that further injury causes slightly greater increases in NT4 compared with non-exercise controls.

## Introduction

Neurotrophic factors are the family of proteins that includes nerve growth factor (NGF), BDNF, NT-3, and NT-4 [1]–[3]. Each neurotrophic factor shows specific selective biological activity, interacting with different members of the tyrosine kinase (trk) receptors [1]. BDNF, which is one of the most active substances to stimulate neurogenesis, acts with tyrosine kinase B (trkB). Neurotrophin 4 (NT-4), which is also called neurotrophin 4 or 4/5 (NT-4 or NT-4/5), also initiates signals by binding with trkB. Since both BDNF and NT-4 bind trkB, the roles of NT-4 and BDNF might be similar. For example, NT-4 might play a role in long-term potentiation and plasticity [4], [5]. BDNF has been frequently described in damaged brain or in response to physiologic stimuli [6]–[8]. The BDNF binding trkB also interacts with NT-4, which indicates that altered expression of trkB can possibly affect the function of NT-4. However, in comparison with BDNF, reports on NT-4 in damaged brain or in response to the physiologic stimuli are rare [9], [10]. Chan et al. showed that treatment with NT-4 reduced the infarction volume in a permanent focal cerebral ischemic rat model [11], demonstrating that NT-4 is involved in ischemic brain injury.

Exercise improves functional recovery following brain injury. It also increases neurotrophic factors, stimulates neurogenesis, or improves resistance to neuronal injury [12]–[14]. Moreover, exercise regenerates and develops the neural system and improves activities associated with learning ability [15]–[17]. Following focal cerebral ischemia, expression of BDNF/trkB and NGF/trkA have been reported, and exercise increases the expression of BDNF/trkB and trkA; it does so more in the side contralateral to the ischemic lesion [6], [18].

Cerebral injury and exercise may alter neurotrophic factors that improve neural function. However, even expression profiles of NT-4 in the ischemic brain are unknown. Therefore, we observed the changes in NT-4 and trkB expression following ischemic injury in the rat brain. We also postulated that exercise can affect the expressions of NT-4 and its receptor, trkB.

## Materials and Methods

### Experimental design and classification of animals

**Figure 1:**
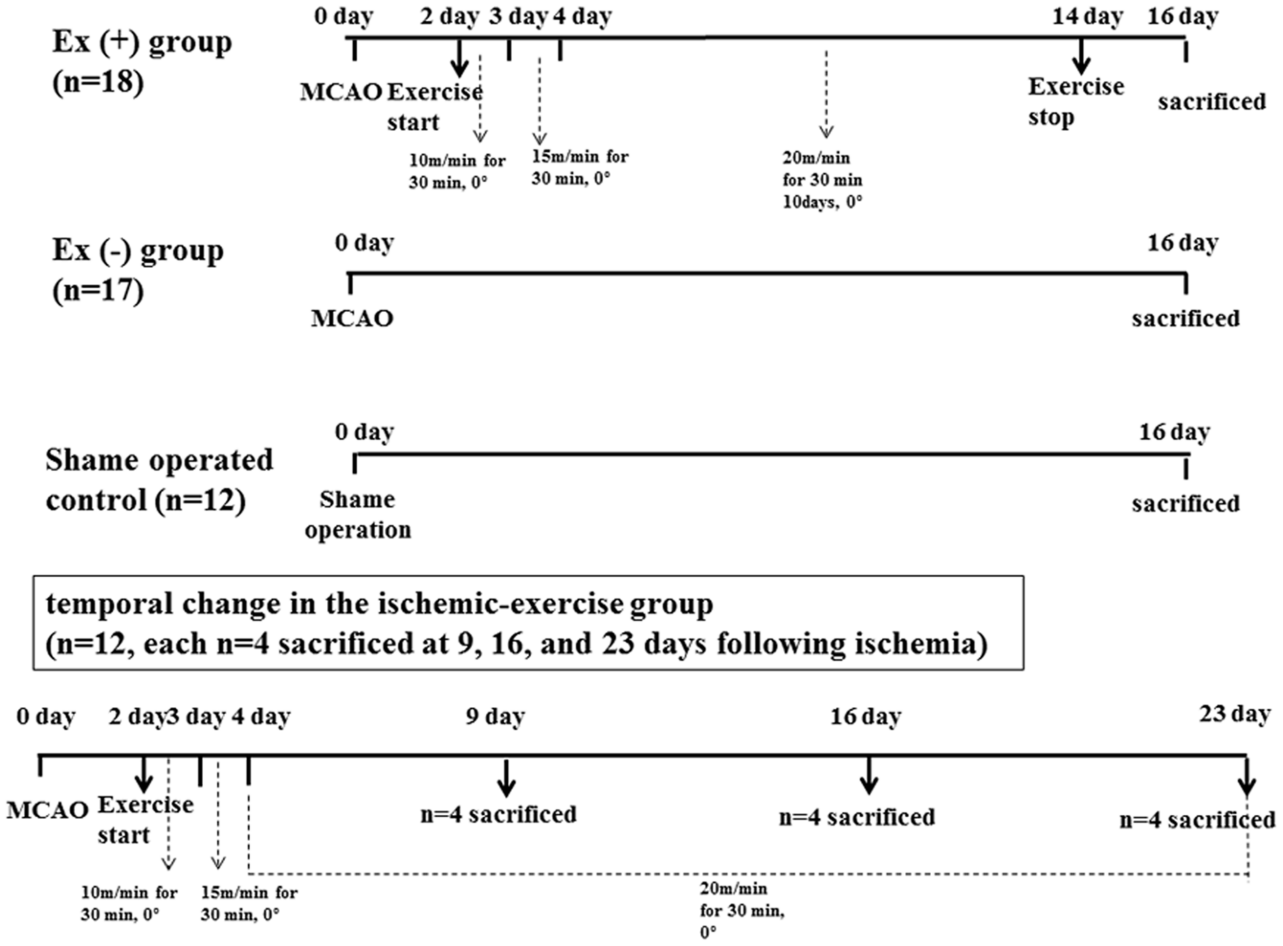
Experimental design. Among total of 59 rats, 35 rats underwent middle cerebral artery occlusion (MCAO), and 12 rats were used as sham-operated control. In 48 hours, the MCAO group was divided into either the exercise (n = 18) or non-exercise group (n = 17). Twelve rats were additionally used for determination of temporal change in the ischemic-exercise group (n = 12, n = 4 each and sacrificed at 9, 16, and 23 days following ischemia).

Experimental conditions and classification of animals for the expression of NT-4 and trkB were the same as previously designed [18]. In brief, a total of 59 adult male Sprague-Dawley rats (275–325 g) were used. Among them, 35 rats underwent middle cerebral artery occlusion (MCAO), and 12 rats were used as sham-operated control. In 48 hours, the MCAO group was divided into either the exercise (n = 18) or non-exercise group (n = 17). The severity was determined according to the Garcia scale as previously described [6], [18]. Six items (spontaneous activity, symmetry of movements, symmetry of forelimbs, climbing the wall of wire cage, reaction to touch, and response to vibrissae touch) were measured with a total score that ranged from 3 to 18. The higher the score, the better the performance: mild (scores 12–18), moderate (8–11), and severe degrees (3–7). Twelve adult male Sprague-Dawley rats (275–325 g) were additionally used for determination of temporal change in the ischemic-exercise group (n = 12, n = 4 each and sacrificed at 9, 16, and 23 days following ischemia) (Figure 1). Protocols for care and use of animals in this procedure were in compliance with guidelines and were approved by the Catholic University animal care committee.

### Surgical procedures

For a focal cerebral ischemia model, modified Longa's method was used, as previously described [19]. Induction was performed using a mixture of 3% isoflurane in 30% O_2_ and 70% N_2_O. For maintenance, 1.5% isoflurane was used. Through a midline cervical incision, the left common carotid artery was exposed at its bifurcation. Branches from the external carotid artery were coagulated. The pterygopalatine artery was ligated with a 5.0 silk suture. A 4.0 nylon monofilament was used for the occlusion. Heating to the tip of this filament made it rounded, and then it was inserted into the bifurcation site of the common carotid artery. The monofilament was advanced 16–18 mm into the internal carotid artery from the bifurcation site in order to occlude vessels at the location of the origins of the middle cerebral and proximal anterior cerebral artery. The monofilament was secured in place with a ligature, and the wound was closed. With this procedure, the origin of the middle cerebral artery was occluded. The animals were allowed to survive with food and water ad libitum. Body temperature was maintained at 37±1°C (rectal temperature) using a thermistor-controlled heating blanket [6], [18].

### Treadmill exercise

For the exercise, we used the treadmill test (Columbus instruments, USA). The start of treadmill exercise was performed 2 days following the MCAO operation. This exercise training was done for 30 min every day for 12 days. The velocity of the treadmill was gradually increased, from 10 m/min on the first exercise day, 15 m/min on the second, and 20 m/min on the third and subsequent days. The tilting angle of the exercise table was maintained and set to 0° [6], [18].

### Immunohistochemistry

On postoperative day 16, for sacrificing, rats were anesthetized, and transcardiac perfusion with heparinized saline followed by 4% paraformaldehyde in phosphate-buffered saline (PBS) was performed. Using a sliding microtome, sections were cut at 30 um thickness. Blocking was done in a mixture of 10% normal goat serum (NGS), 1% bovine serum albumin (BSA), 0.2% Triton X-100, and 1% H_2_O_2_ in PBS. After washing with PBS three times, primary antibody was incubated in 10% NGS and 1% BSA for 40 h at 4°C. For detection of NT-4 and trkB immunoreactivity, anti-NT-4 (1∶300, Santa Cruz, CA, USA) and anti-trkB (1∶300, Santa Cruz, CA, USA) antibodies were used. Immunoperoxidase labeling was performed using a DAB kit (Dako, Carpinteria, CA), and slides were evaluated using an Olympus BX51 microscope (Olympus, Japan) [6], [18].

### Western blot analysis

For protein extraction, brains were dissected into right and left hemispheres and placed on ice in 10 volumes of cold homogenization buffer (50 mM Tris, 120 mM NaCl, pH 7.4). Protease inhibitors (Complete Mini, Gibco, Grand Island, NY, USA) were added, and then the tissue was homogenized. Protein concentrations were determined by Bradford method (Bio-Rad, Richmond, CA, USA). For each well, 20 µg of protein extracts were loaded and separated by sodium dodecyl sulfate-polyacrylamide gel electrophoresis using 10% polyacrylamide with 0.05% bis-acrylamide [20]. Proteins were transferred to nitrocellulose membrane and probed with anti-NT-4 (1∶300, Santa Cruz, CA, USA) and anti-trkB (1∶300, Santa Cruz, CA, USA). Peroxidase anti-rabbit IgG (vector, PI-1000, 1∶3000 dilution) was used as a secondary antibody. β tubulin (1∶300, Santa Cruz, CA, USA) was used for an internal control. Signals were detected by enhanced chemiluminescence (Supersignal, Pierce, Rockford, IN, USA) using autoradiograms exposed from 10 to 30 min [6], [18]. These experiments were repeated independently in triplicate.

### Statistical analysis

The Mann-Whitney test was used to compare the control and exercise groups. In addition, non-parametric test for the paired sample was also performed. SPSS ver. 12.0 was used, and a p-value below 0.05 was considered to be statistically significant. We replicated experiments more than three times to confirm the results.

## Results

### Expression profile of NT-4

**Figure 2:**
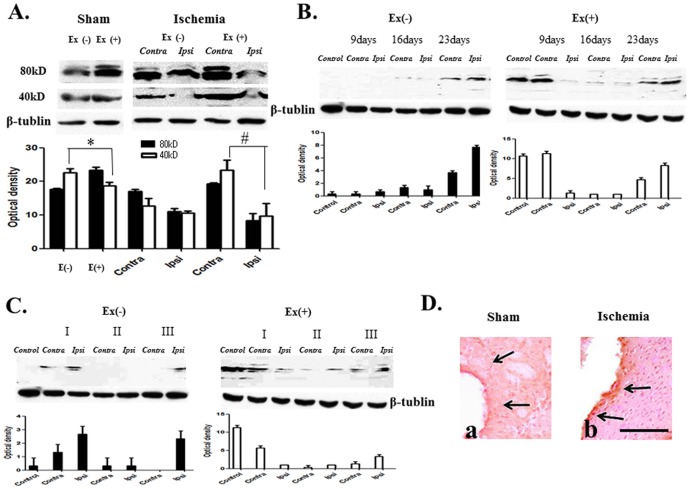
NT-4 expression profile. (**A**) Two forms, dimer (80 kDa) and monomer (40–47 kDa), were detected. Ischemia decreases monomer and dimer proteins in the ipsilateral region (Ipsi). Exercise increased monomer and dimer proteins in both hemispheres, particularly in the contralateral hemisphere in ischemia (# p<0.05, n = 7). Exercise-only increased dimer (* p<0.05, n = 7). (**B**) Expression of dimers increased at postinfarct day 23 in ischemia. Exercise increased dimer proteins at postinfarct as early as day 9 and particularly in the contralateral hemisphere at postinfarct day 23. (p<0.05, n = 6). (**C**) Dimer level is lowered in moderate to severe conditions. Exercise induces increased expression of dimers, more so in the milder condition (I: mild, II: moderate, III: severe) (p<0.05, n = 5). (**D**) The distribution of immunoreactivity by exercise increased adjacent to the ischemic region (b) comparing to the ischemia-only control (a). ▄ = 100 um.

NT-4 exists in two forms, either as a dimer (80 kDa) or as a monomer (40–47 kDa). Both forms of proteins were decreased in the ipsilateral ischemic region at 2 weeks when compared to the non-ischemic contralateral side (Figure 2A). NT-4 was increased by treadmill exercise, more so in the contralateral hemisphere following ischemic injury. Exercise alone increased monomer and dimer forms of NT-4 proteins in the bilateral hemispheres (Figure 2A). Analysis of temporal changes in NT-4 showed that NT-4 dimer protein, the level of which was low in week 2, increased post-infarct on day 23. Treadmill exercise increased NT-4 as early as post-infarct day 9. At post-infarct day 23, this dimer protein was also increased, particularly in the contralateral hemisphere (Figure 2B). NT-4 dimer protein decreased when the ischemic severity increased. Exercise increased the expression of NT-4 dimer protein (Figure 2C). NT-4 showed that immunoreactivity increased in the ischemic region, and the distribution of immunoreactivity came out adjacent to the ischemic region after exercise (Figure 2D).

### Expression profiles of trkB

**Figure 3:**
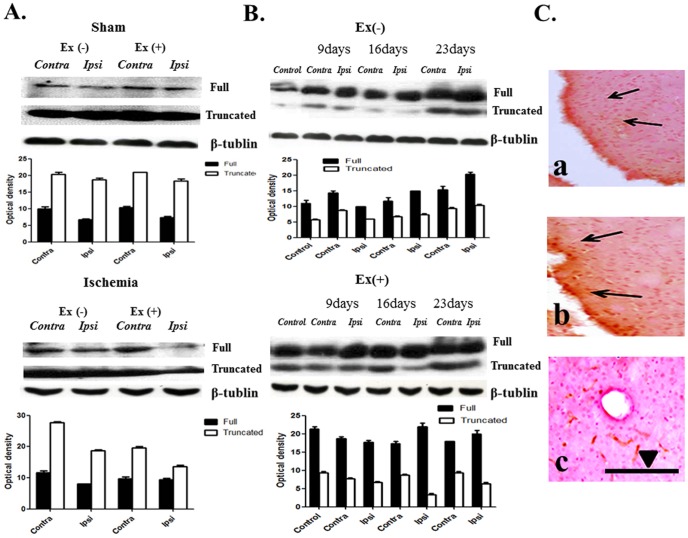
TrkB expression. (**A**) Two forms of trkB are noted: full length form (140 kDa) and truncated form (90∼95 kDa). Ischemia decreased the full-length protein in the ipsilateral region (Ipsi). Exercise increased two forms of the protein in both hemispheres, particularly contralateral (Contra) to the ischemic hemisphere (p<0.05, n = 7). (**B**) Expression of two forms of protein increased at day 23 after ischemia. Exercise increased the full-length form in both hemispheres at day 16 and increased the truncated form by day 16, particularly in the contralateral hemisphere (p<0.05, n = 6). (**C**) (a) Immunoreactivities in the ischemic region. (b) Exercise increased the immunoreactivities adjacent to the ischemic region in the ipsilateral hemisphere. (c) In the control hemisphere, exercise increased immunoreactivities, particularly in vascular structures. ▄ = 100 um.

TrkB exists in two forms, either as a full-length (140 kDa) protein or as a truncated (90–95 kDa) protein. In the ischemia group, the full-length protein was decreased; however, the truncated protein was not changed. Exercise increased the full length and truncated proteins in ischemic conditions. Treadmill exercise also increased the full-length protein in both hemispheres of the sham control (Figure 3A).

Temporal changes in trkB showed that expression of the two forms of trkB increased following day 23. After exercise, expression of the full-length form was increased in both hemispheres at day 16, and the truncated form was increased by day 16, particularly in the contralateral hemisphere (Figure 3B). No relationship between the expression of trkB protein and severity of ischemia was observed.

Immunohistochemistry showed that exercise increased immunoreactivity in both hemispheres of the sham group, particularly in vascular structures, and exercise also increased the distribution of immunoreactivity around the ischemic region in the ipsilateral hemisphere (Figure 3C).

## Discussion

In previous studies, we confirmed the expression of BDNF/trkB and NGF/trkA following focal cerebral ischemia [6],[18].

In this study, we attempted to observe the changes in NT-4 and trkB expression following ischemic injury in the rat brain. We hypothesized that exercise changes expression of neurotrophic factors and their tyrosine kinase receptors. Our results showed that ischemia decreased NT-4 and trkB, a specific receptor of the NT-4 full-length protein, in the ipsilateral region; however, there were no changes in the truncated protein. Treadmill exercise altered the levels of NT-4 and trkB, increasing them more in the contralateral hemisphere. In terms of immunohistochemistry, immunoreactivities of NT-4 and trkB appeared to be most predominant around the ischemic area. These staining intensities became dense and smaller following exercise. These results suggest that NT-4 altered in response to ischemic injury, and treadmill exercise plays a role in the changes of neurotrophins and their receptors.

Although NT4 is included in the neurotrophic family, NT-4 and trkB decreased, and their expressions in the ischemic brain were different. Since NT-4 has a high affinity for trkB, the function of NT-4 is supposed to be the same as BDNF, which also plays a role in long-term potentiation and plasticity [4], [5]. However, BDNF in ischemic rat brain increased [6] whereas NT-4 decreased in the same experimental set in this study. These findings cannot account for the fact that the roles of NT-4 and BDNF are identical.

Effects of exercise include a better functional outcome [6], [18], exercise may increase neurotrophic factors, neurogenesis, or have neuroprotective effects [12]–[14]. Duration and intensity of exercise are factors for promoting plasticity and enhancement of performance. Compared with voluntary exercise, progressive treadmill exercise was intense and lasted long enough to improve brain function [6], [8], [14], [21], [22]. As a result, treadmill exercise enhanced NT-4 in the contralateral hemisphere in an ischemic model and even in control sham-operated rats. This supports idea that increasing neurotrophic factors contribute to functional recovery [12], [13]. Immunohistochemistry showed more NT-4 immunoreactivity in the ischemic area compared to the non-ischemic region. Exercise concentrated the area of immunoreactivities in our experiment. It has been reported that exercise reduces brain damage in ischemic rats [23], suggesting one possibility that accounts for the concentrating area of immunoreactivities. Immunohistochemistry also showed that exercise increased trkB immunoreactivity, particularly in vascular structures. Exercise is known to be associated with regional angiogenesis [23]. There is no direct evidence to show that trk receptors increased in vascular structures.

Trials for treatment with neurotrophic factors involving direct administration under pathologic conditions have been conducted [5], [24], [25]. Among types of brain injury, stroke is the most common cause that leads to death [26]. Studies of exercise as a rehabilitation program show that it can also change neurotrophic factors and trk receptors in the damaged brain [6], [14], [27]. Under experimental cerebral ischemia and exercise conditions, the expression profiles of NT4/trkB as well as NGF/trkA and BDNF/trkB are changed [6], [18]. Taken together, these findings suggest that functional recovery in cerebral ischemia is associated with not only BDNF or NGF, but it can also be mediated by NT-4 and other tyrosine kinase receptors.

## Conclusions

Overall, ischemia decreased NT-4 and trkB expressions in a permanent middle cerebral artery occlusion rat model. However, treadmill exercise changed expressions of NT-4 and trkB. Altered expression profiles in ischemic brain indicate that NT-4 and trkB might participate in the recovery process in rats with brain damage.
